# Noise-Enhanced Hamming Code Transmission over an Additive Gaussian Mixture Noise Channel with One-Bit ADCs

**DOI:** 10.3390/s26175597

**Published:** 2026-09-03

**Authors:** Qiqing Zhai, Youguo Wang

**Affiliations:** College of Science, Nanjing University of Posts and Telecommunications, Nanjing 210023, China; zhaiqq@njupt.edu.cn

**Keywords:** stochastic resonance, Hamming codes, additive Gaussian mixture noise channel, one-bit analog-to-digital converter, speech signal transmission

## Abstract

Random noise may have a positive effect on the transmission and reception of signals in some nonlinear communication systems, which is termed as stochastic resonance (SR) or noise-enhanced effect. In this paper, we investigate the performance of signals transmitted over an additive Gaussian mixture noise channel. At the receiving end, before detection by a maximum likelihood (ML) detector, signals are injected with an intentionally added Gaussian noise and quantized by one-bit analog-to-digital converters (ADCs). How the injected Gaussian noise affects the transmission performance and whether the SR effect exists are explored. For both the uncoded signal and Hamming-coded signal, the error probabilities of transmission are derived. The corresponding performance is shown theoretically and by simulation. We find that under certain conditions, the injected Gaussian noise may improve the performance of the signal transmission, which indicates that SR exists in this communication system. In particular, the optimal noise intensity is determined and the minimum error probability is achieved. Moreover, the transmission of a speech signal is presented as a specific example, in which the noise-enhanced effect is demonstrated as well.

## 1. Introduction

The noise-enhanced effect, sometimes called stochastic resonance (SR), has been widely discussed in many fields such as signal detection [1], channel quantization [2], machine learning [3], fault detection [4,5], and so on. It becomes a common view that random noise can be used to improve the performance of some nonlinear systems [6]. In particular, when the channel or the receiver is nonlinear, some injected noise may play a positive role in information transmission. Sugiura et al. considered the transmission of serially concatenated turbo codes contaminated by Gaussian mixture noise [7]. They used a single comparator to quantize the received signal and then carried out the iterative decoding. In that scenario, a low bit error rate was achieved with the help of noise. Based on the study in [7], we employed another nonlinear system, namely the array of threshold units, and discussed the SR effect of noise-enhanced turbo decoding [8]. For low-density parity-check (LDPC) codes and quantum LDPC codes, it has also been shown that noisy decoders perform better than the noiseless ones, since random noise may help decoders escape from trapping sets [9,10].

Apart from the above results, few SR techniques have been applied to error correction coding. It is worthwhile to further investigate the SR benefits in the transmission of other error-correcting codes. In this paper, we pay attention to the performance of Hamming codes transmitted over an additive Gaussian mixture noise channel and quantized by one-bit analog-to-digital converters (ADCs). Both encoding and decoding of Hamming codes entail low complexity. Moreover, the weight distribution of Hamming codes is known in closed form, which allows us to theoretically derive the transmission performance of Hamming codes and explore the noise-enhanced effect.

### 1.1. Related Work

The Hamming code family is an important class of error-correcting codes [11]. It is able to correct one error in a block of symbols. In some communication systems, e.g., the filtered OFDM system, Hamming codes are used to improve system performance [12]. When more errors were introduced in the Hamming codeword, Rurik and Mazumdar investigated the average number of errors in the decoded message [13]. By using different decoding methods, they showed the error-reducing capacities of Hamming codes. Moreover, due to the error-correcting property, Hamming codes can be used for data hiding and data extracting in image or video steganography [14,15].

In many communication systems, noise is not always Gaussian [16]. Particularly, Gaussian mixture noise can be used to model the interference in some real systems, such as underwater acoustic communication systems, CDMA communication systems, and ultra-wideband communication systems [17,18,19]. Motivated by these studies, we focus on an additive Gaussian mixture noise channel in this paper. In the SR literature, the noise-enhanced effect has been extensively studied when system noise follows a Gaussian mixture distribution. Kay investigated the detection of a direct current (DC) signal embedded in a Gaussian mixture noise and showed that the detectability of a suboptimal detector can be improved by adding an independent Gaussian noise [20]. Even for the optimal detector, Chapeau-Blondeau and Rousseau demonstrated that, under certain conditions, the probability of a detection error may decrease as the Gaussian mixture noise level increases [21]. Moreover, Akbay and Gezici discovered the noise benefits in joint detection and estimation systems as well [22].

At the receiving end of a communication system, an ADC is often used to sample and quantize the received analog signal. Due to the characteristics of a high sampling rate and low energy consumption, low-resolution (1–3 bits) ADCs have drawn much attention [23,24]. As a special low-resolution ADC, the one-bit ADC has also been widely investigated for wireless communications [25,26]. In particular, the one-bit ADC can be simply formed by a comparator with a threshold level. Such a comparator (or an array of comparators) exhibits nonlinear behaviors. As a result, the SR effect may exist in the ADCs [27,28].

### 1.2. Contributions and Outline

- We focus on an additive Gaussian mixture noise channel in this paper. As an uncoded binary phase shift keying (BPSK) signal is transmitted over the channel, one-bit ADCs and a maximum likelihood (ML) detector are designed to detect the output of the channel. In the uncoded scenario, the bit error probability is derived.
- Before being transmitted, the signal is encoded by the Hamming coding scheme. At the receiving end, the syndrome decoding is adopted to detect errors. In this case, the codeword error probability and a lower bound of the information bit error probability are determined. At the same time, the SR effect is demonstrated.
- As a specific example, a speech (audio) signal transmitted over the additive Gaussian mixture noise channel is considered. Correspondingly, the performance of noise enhancing speech transmission is displayed as well.

The rest of this paper is organized as follows. The model of information transmission is depicted in Section 2. Based on this model, noise-enhanced performance of information transmission in the uncoded case and the Hamming coding case is presented in Section 3 and Section 4, respectively. An example of speech signal transmission is given in Section 5 to show the noise-enhanced effect. At last, conclusions are described in Section 6.

## 2. The Model of Information Transmission

**Figure 1:**
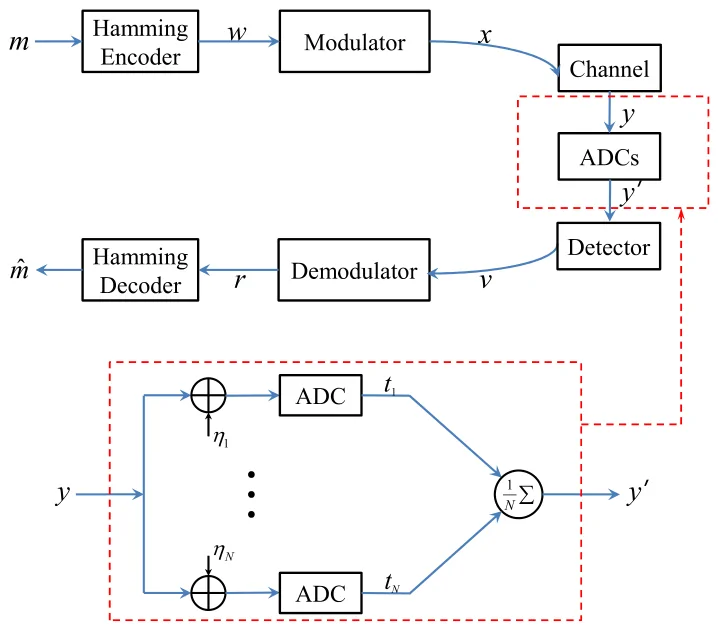
The model of a communication system.

The model of a communication system is shown in Figure 1. Unless otherwise specified, all variables here denote scale elements.

The source information to be transmitted is a binary signal, i.e., m∈{0,1}, with the a priori probabilities $P_{0}$ and $P_{1}$ ($P_{0}+P_{1}=1$). At first, blocks of information bits are encoded by a Hamming encoder. And then, *w*, the bit of Hamming codewords, is mapped into a BPSK symbol *x*, where x∈{−1,+1}.

As the symbol *x* is transmitted through the channel, it will be corrupted by the channel noise ξ. We assume that the channel is slow-varying; that is, the channel noise parameters remain constant over some time interval. Moreover, we focus on a kind of additive Gaussian mixture noise, whose distribution is a linear combination of two Gaussian terms. Specifically, the noise’s probability density function (PDF) is

(1) $$p_{\xi }\left(\xi \right)=\frac{1}{2}N\left(\xi ;\mu_{\xi },\sigma_{\xi }^{2}\right)+\frac{1}{2}N\left(\xi ;-\mu_{\xi },\sigma_{\xi }^{2}\right),$$

where $N\left(\xi ;\mu ,\sigma^{2}\right)=\frac{1}{\sqrt{2\pi }\sigma }\exp\left[-\frac{{\left(\xi -\mu \right)}^{2}}{2\sigma^{2}}\right]$ and $\mu_{\xi }>0$. The noise distribution in Equation (1) can be extended to a more general case involving more Gaussian terms. Theoretically, any non-Gaussian noise distribution can be approximated by a finite sum of Gaussian probability density functions [29]. However, the mixing coefficient and Gaussian variance may vary from one term to another. Fortunately, there are a variety of approaches to estimate these Gaussian mixture parameters, e.g., the expectation maximization (EM) algorithm. In this paper, we just pay attention to the noise expressed by Equation (1). For a general Gaussian mixture noise channel, similar results may be achieved.

At the receiver, the channel output *y* is detected by the ADCs and an ML detector. As shown in Figure 1, there is a parallel array consisting of *N* identical one-bit ADCs, each of which has a threshold *u*. Before being quantized by these ADCs, *y* is mixed with some noises $\eta_{i}$ (i=1,2,⋯,N). Such intentionally injected noises are expected to induce an SR effect and enhance information transmission and reception. Following Refs. [30,31], we assume that noises $\eta_{i}$ (i=1,2,⋯,N) are mutually independent and have an identical PDF $p_{\eta }\left(\eta \right)$. In this paper, a Gaussian PDF with zero mean is considered. That is to say,

$$p_{\eta }\left(\eta \right)=N\left(\eta ;0,\sigma^{2}\right),$$

where σ is the standard deviation or intensity of the Gaussian noise. In practice, the noise can be generated by some hardware Gaussian noise generator [32]. It is equally important to examine the SR effect when the injected Gaussian noises $\eta_{i}$ (i=1,2,⋯,N) are corrected, which will be investigated in the future. Moreover, the noises $\eta_{i}$ (i=1,2,⋯,N) are independent of the channel noise ξ.

The ADCs and the ML detector produce the symbol v∈{−1,+1}. And then *v* is demodulated and the binary bit *r* (r∈{0,1}) is obtained. Finally, the estimation of source information, denoted by $\hat{m}$, can be obtained by using a Hamming decoding technique. The performance of information transmission is determined by comparing the original and the decoded information bits.

## 3. Transmission Performance of an Uncoded Signal

To analytically study the performance of Hamming coding, the transition probability of *r* given *w* and the error probability Pr{w≠r} are needed. So first, we directly focus on the transmission of signal *w*, which can be seen as an uncoded case. We assume that the binary symbol *w* has the same distribution as *m*, i.e.,

$$\mathrm{Pr}\left\{x=-1\right\}=\mathrm{Pr}\left\{w=0\right\}=P_{0},$$

$$\mathrm{Pr}\left\{x=+1\right\}=\mathrm{Pr}\left\{w=1\right\}=P_{1}.$$

As the BPSK symbol *x* (x∈{−1,+1}) is transmitted through the additive Gaussian mixture noise channel, the channel output y=x+ξ has a conditional PDF

(2) $$p_{y}\left(y|x\right)=\frac{1}{2}N\left(y;\mu_{\xi }+x,\sigma_{\xi }^{2}\right)+\frac{1}{2}N\left(y;-\mu_{\xi }+x,\sigma_{\xi }^{2}\right).$$

For each one-bit ADC characterized by a threshold *u*, the quantized output is determined as

(3) $$t_{i}=\left\{\begin{array}{cc} 0, & y+\eta_{i}\leq u\\ 1, & y+\eta_{i}>u \end{array}\right..$$

Due to symmetry, we will set u=0 in this paper. By averaging all $t_{i}$’s, we get $y^{\prime }=\frac{1}{N}\sum_{i=1}^{N}t_{i}$. Obviously, $y^{\prime }\in A=\left\{0,\frac{1}{N},\frac{2}{N},\cdots ,1\right\}$.

According to the PDF of the injected noise (i.e., $p_{\eta }\left(\eta \right)$), we have

$$\begin{array}{cc} \mathrm{Pr}\{t_{i}=1|y\} & =\mathrm{Pr}\left\{\eta_{i}>u-y\right\}=\int_{u-y}^{+\infty }p_{\eta }\left(\eta \right)d\eta =Q\left(\frac{u-y}{\sigma }\right)≜q\left(y\right),\\ \mathrm{Pr}\{t_{i}=0|y\} & =\mathrm{Pr}\left\{\eta_{i}\leq u-y\right\}=\int_{-\infty }^{u-y}p_{\eta }\left(\eta \right)d\eta =1-q\left(y\right), \end{array}$$

where $Q\left(z\right)=\int_{z}^{+\infty }\frac{1}{\sqrt{2\pi }}\exp\left\{-\frac{t^{2}}{2}\right\}dt$. Furthermore, for j=0,1,⋯,N, the conditional probability of $y^{\prime }$ given *y* can be calculated as follows:

(4) Pry′=jN|y=Njq(y)j1−q(y)N−j.

Then we have

(5) Pry′=jN|x=∫−∞+∞Pry′=jN|ypy(y|x)dy=∫−∞+∞Njq(y)j1−q(y)N−jpy(y|x)dy,

where $p_{y}\left(y|x\right)$ is given by Equation (2), and x∈{−1,+1}.

Based on $y^{\prime }$, the log likelihood ratio (LLR) is defined as

$$L\left(y^{\prime }\right)=\ln\frac{\mathrm{Pr}\{y^{\prime }|x=+1\}}{\mathrm{Pr}\{y^{\prime }|x=-1\}}.$$

More explicitly, by using Equation (5), for each $y^{\prime }=\frac{j}{N}\in A$, the LLR can be expressed as

(6) $$L\left(y^{\prime }=\frac{j}{N}\right)=\ln\frac{\int_{-\infty }^{+\infty }{q(y)}^{j}{\left(1-q(y)\right)}^{N-j}p_{y}\left(y|x=+1\right)dy}{\int_{-\infty }^{+\infty }{q(y)}^{j}{\left(1-q(y)\right)}^{N-j}p_{y}\left(y|x=-1\right)dy}.$$

Then the ML detector may make a decision depending on Equation (6). That is,

(7) $$v=\left\{\begin{array}{cc} -1, & L(y^{\prime })\leq 0\\ +1, & L(y^{\prime })>0 \end{array}\right.,$$

or equivalently, $r=\left\{\begin{array}{cc} 0, & L(y^{\prime })\leq 0\\ 1, & L(y^{\prime })>0 \end{array}\right..$

Let $A_{1}=\left\{y^{\prime }|L\left(y^{\prime }\right)>0,y^{\prime }\in A\right\}$ and $A_{0}=\left\{y^{\prime }|L\left(y^{\prime }\right)\leq 0,y^{\prime }\in A\right\}$. It is clear that $A_{0}\cup A_{1}=A$. Consequently, the transition probabilities are

(8) P1|0=Pr{r=1|w=0}=∑{j|jN∈A1}∫−∞+∞Njq(y)j1−q(y)N−jpy(y|x=−1)dy,

(9) P0|1=Pr{r=0|w=1}=∑{j|jN∈A0}∫−∞+∞Njq(y)j1−q(y)N−jpy(y|x=+1)dy.

According to Equations (8) and (9), the bit error probability in the uncoded scenario can be expressed as follows:

(10) $$P_{e}=P_{0}P_{1|0}+P_{1}P_{0|1}.$$

### 3.1. The Case of N=1

When just one ADC is employed, the bit error probability determined by Equation (10) has a specific version, as shown below:

(11) $$\begin{array}{cc} P_{e}= & P_{0}\cdot \frac{1}{2}\left[Q\left(\frac{u+1-\mu_{\xi }}{\sqrt{\sigma_{\xi }^{2}+\sigma^{2}}}\right)+Q\left(\frac{u+1+\mu_{\xi }}{\sqrt{\sigma_{\xi }^{2}+\sigma^{2}}}\right)\right]\\ \; & +P_{1}\cdot \left[1-\frac{1}{2}Q\left(\frac{u-1-\mu_{\xi }}{\sqrt{\sigma_{\xi }^{2}+\sigma^{2}}}\right)-\frac{1}{2}Q\left(\frac{u-1+\mu_{\xi }}{\sqrt{\sigma_{\xi }^{2}+\sigma^{2}}}\right)\right], \end{array}$$

where $\mu_{\xi }$ and $\sigma_{\xi }$ are parameters of the channel noise, and σ is the intensity of the intentionally injected Gaussian noise. Particularly, for u=0, Equation (11) can be further simplified as

(12) $$P_{e}=\frac{1}{2}\left[Q\left(\frac{1+\mu_{\xi }}{\sqrt{\sigma_{\xi }^{2}+\sigma^{2}}}\right)+Q\left(\frac{1-\mu_{\xi }}{\sqrt{\sigma_{\xi }^{2}+\sigma^{2}}}\right)\right].$$

Since the bit error probability $P_{e}$ is closely related to the injected noise intensity σ, we may make use of the noise-enhanced effect and obtain a small $P_{e}$ by adjusting the parameter σ. The following lemma gives a sufficient condition for the existence of the noise-enhanced effect (i.e., SR).

**Lemma 1.**  *For N=1, when the following Equation (13) is satisfied, the optimal intensity $\sigma_{\mathrm{opt}}$ of the injected Gaussian noise, which induces the minimum $P_{e}$, is nonzero.*

(13) $$\begin{array}{cc} \; & P_{0}\left[\left(u+1-\mu_{\xi }\right)\exp\left(-\frac{{\left(u+1-\mu_{\xi }\right)}^{2}}{2\sigma_{\xi }^{2}}\right)+\left(u+1+\mu_{\xi }\right)\exp\left(-\frac{{\left(u+1+\mu_{\xi }\right)}^{2}}{2\sigma_{\xi }^{2}}\right)\right]\\ < & P_{1}\left[\left(u-1-\mu_{\xi }\right)\exp\left(-\frac{{\left(u-1-\mu_{\xi }\right)}^{2}}{2\sigma_{\xi }^{2}}\right)+\left(u-1+\mu_{\xi }\right)\exp\left(-\frac{{\left(u-1+\mu_{\xi }\right)}^{2}}{2\sigma_{\xi }^{2}}\right)\right]. \end{array}$$

The proof is in Appendix A.

More specifically, the threshold u=0 is considered, based on which the optimal parameter of the intentionally injected noise can be theoretically determined.

**Theorem 1.**  *For u=0 and N=1, the optimal variance of the injected Gaussian noise is $\sigma_{\mathrm{opt}}^{2}=0$ if $0<\mu_{\xi }\leq 1$; $\sigma_{\mathrm{opt}}^{2}={\left[\frac{2\mu_{\xi }}{\ln\frac{\mu_{\xi }+1}{\mu_{\xi }-1}}-\sigma_{\xi }^{2}\right]}^{+}$ if $\mu_{\xi }>1$. Here, ${\left[z\right]}^{+}=\max\left\{z,0\right\}$.*

The proof is in Appendix B.

**Remark 1.**  *Theorem 1 indicates a necessary and sufficient condition for SR to occur, i.e., the channel noise having the PDF in Equation (1) should satisfy $\mu_{\xi }>1$ and $\sigma_{\xi }^{2}<2\mu_{\xi }/\ln\frac{\mu_{\xi }+1}{\mu_{\xi }-1}$. Once the parameters $\mu_{\xi }$ and $\sigma_{\xi }^{2}$ are estimated, we may decide whether to add Gaussian noise into the received signal y, and how strong the added Gaussian noise should be.*

When $\mu_{\xi }$ is comparatively small ($\mu_{\xi }\to 0$), the Gaussian mixture noise channel will be approximated as an additive white Gaussian noise (AWGN) channel. According to Theorem 1, when u=0, the optimal σ is zero and any additionally introduced Gaussian noise will always increase the bit error probability $P_{e}$. It means that no SR effect exists in this case. However, when the threshold u≠0, SR may also occur. The discussion is presented in Appendix C.

When $\sigma_{\xi }^{2}$ is comparatively large, any injected Gaussian noise will deteriorate the transmission performance as well. For such a bad channel (having a strong channel noise), it is almost impossible to improve the performance by exploiting the SR effect.

**Figure 2:**
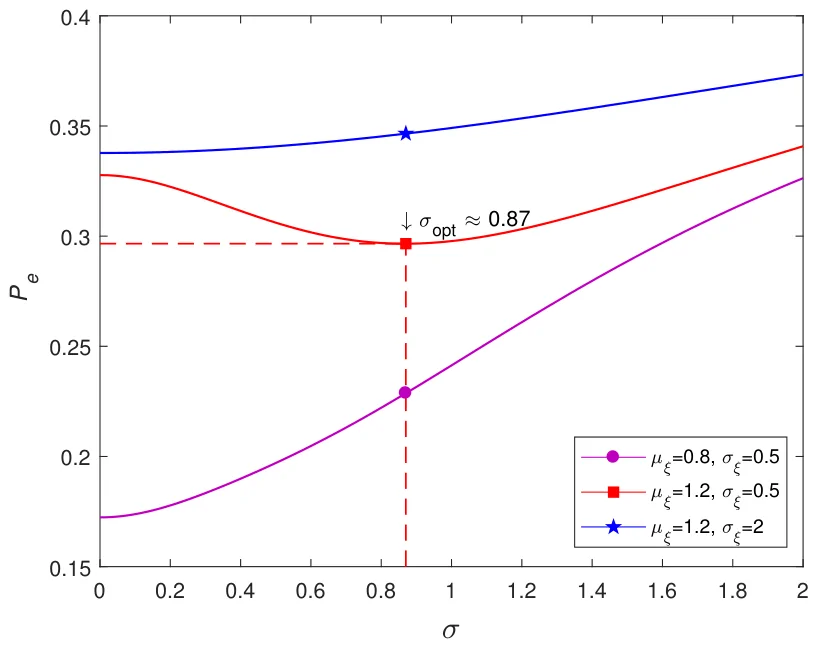
The bit error probability $P_{e}$ versus the intentionally injected Gaussian noise intensity σ. Here, the number of one-bit ADCs is N=1, and the threshold of the ADC is u=0. The probabilities of the input signal are $P_{0}=P_{1}=0.5$.

Under certain channel conditions, Figure 2 shows the performance induced by the injected Gaussian noise.

When the parameters of the channel noise are set to $\mu_{\xi }=0.8$, $\sigma_{\xi }=0.5$ or $\mu_{\xi }=1.2$, $\sigma_{\xi }=2$, the condition in Remark 1 is not satisfied. In the two cases, the bit error probability is a monotonically increasing function of σ. On the other hand, the channel noise with $\mu_{\xi }=1.2$, $\sigma_{\xi }=0.5$ satisfies the condition in Remark 1. In this scenario, a little amount of injected Gaussian noise helps to reduce the bit error probability. As shown in Figure 2, the optimal intensity is $\sigma_{\mathrm{opt}}=\sqrt{2\mu_{\xi }/\ln\frac{\mu_{\xi }+1}{\mu_{\xi }-1}-\sigma_{\xi }^{2}}=\sqrt{\frac{2.4}{\ln11}-0.25}\approx 0.87$.

### 3.2. The Case of N>1

**Figure 3:**
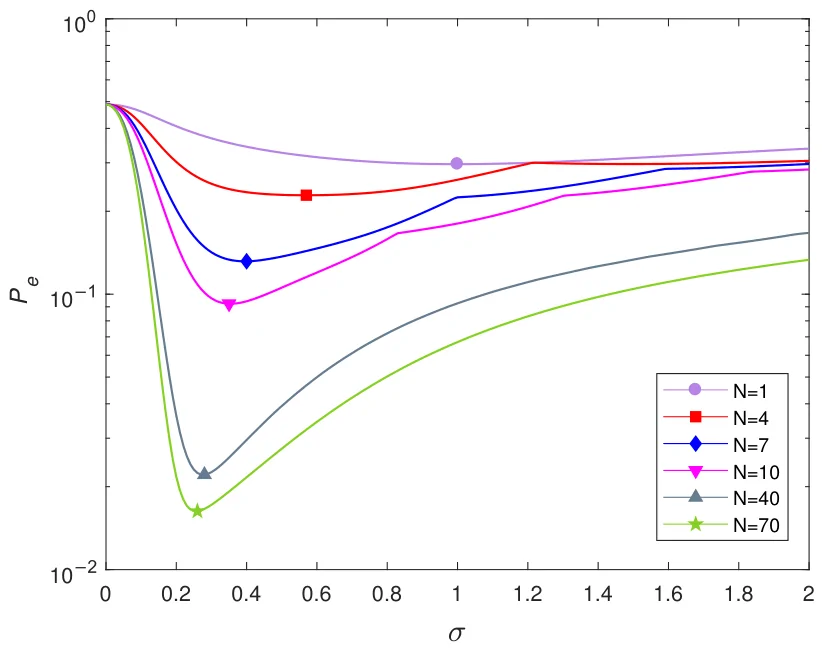
The bit error probability $P_{e}$ versus the intentionally injected Gaussian noise intensity σ in the case of N=1,4,7,10,40, and 70. The channel noise is a Gaussian mixture noise with $\mu_{\xi }=1.2$, $\sigma_{\xi }=0.1$. Moreover, $P_{0}=P_{1}=0.5$ and the threshold of the one-bit ADC is u=0.

When the number of one-bit ADCs is more than one, it is hard to obtain some analytical conclusions. In this subsection, we decide the sets $A_{1}$ and $A_{0}$ and calculate Equations (8) and (9) numerically. Then the performance is obtained according to Equation (10). As the channel noise is predetermined, Figure 3 displays the injected Gaussian noise-induced performance for various *N*’s.

However, it is difficult to analytically determine whether the sufficient condition in Remark 1 holds for N>1. We can also observe the SR effect, as shown in Figure 3, when the condition is satisfied. For each *N*, Gaussian noise helps to enhance the transmission and reception performance. Obviously, the noise-enhanced performance becomes better and better as the number *N* increases. Moreover, the minimum $P_{e}$ is achieved when the optimal noise intensity $\sigma_{\mathrm{opt}}$ is used. In Figure 3, the corresponding point in each curve is marked by the circle, the square, and so on. We find that $\sigma_{\mathrm{opt}}$ gets smaller upon increasing the number *N*.

### 3.3. Sensitivity Analysis

In this subsection, we will explore the performance of the noise-induced ML detector given by Equations (6) and (7), when the parameters of channel noise, i.e., $\mu_{\xi }$ and $\sigma_{\xi }$, are not accurately estimated.

Let ${\hat{\mu }}_{\xi }$ and ${\hat{\sigma }}_{\xi }$ denote the estimates of the two parameters, which should be positive (${\hat{\mu }}_{\xi }>0$ and ${\hat{\sigma }}_{\xi }>0$). Then, the estimated conditional PDF of the channel output *y* given *x* is

(14) $${\hat{p}}_{y}\left(y|x\right)=\frac{1}{2}N\left(y;{\hat{\mu }}_{\xi }+x,{\hat{\sigma }}_{\xi }^{2}\right)+\frac{1}{2}N\left(y;-{\hat{\mu }}_{\xi }+x,{\hat{\sigma }}_{\xi }^{2}\right).$$

Using Equation (14), we may obtain the estimated conditional probability of $y^{\prime }$ given *x* as follows:

Pr^y′=jN|x=∫−∞+∞Njq(y)j1−q(y)N−jp^y(y|x)dy,j=0,1,⋯,N.

According to the estimated conditional probability, the LLR becomes

$$\hat{L}\left(y^{\prime }\right)=\ln\frac{\hat{\mathrm{Pr}}\left\{y^{\prime }|x=+1\right\}}{\hat{\mathrm{Pr}}\left\{y^{\prime }|x=-1\right\}},$$

which can be used to make a decision, as shown in Equation (7). Specifically, we have ${\hat{A}}_{1}=\left\{y^{\prime }|\hat{L}\left(y^{\prime }\right)>0,y^{\prime }\in A\right\}$ and ${\hat{A}}_{0}=\left\{y^{\prime }|\hat{L}\left(y^{\prime }\right)\leq 0,y^{\prime }\in A\right\}$.

In this case, Equations (8) and (9) can be rewritten as

(15) P1|0=∑{j|jN∈A^1}∫−∞+∞Njq(y)j1−q(y)N−jpy(y|x=−1)dy,

(16) P0|1=∑{j|jN∈A^0}∫−∞+∞Njq(y)j1−q(y)N−jpy(y|x=+1)dy.

Consequently, according to Equations (15) and (16), the bit error probability with inaccurate parameter estimates, denoted by ${\hat{P}}_{e}$, can be determined by Equation (10) as well.

In particular, for N=1, a straightforward calculation shows that $\hat{\mathrm{Pr}}\left\{y^{\prime }=0|x=-1\right\}>\hat{\mathrm{Pr}}\left\{y^{\prime }=0|x=+1\right\}$ and $\hat{\mathrm{Pr}}\left\{y^{\prime }=1|x=-1\right\}<\hat{\mathrm{Pr}}\left\{y^{\prime }=1|x=+1\right\}$. Thus, under exact parameter values and inaccurate parameter estimates, the ML detector yields identical decisions, i.e., ${\hat{A}}_{1}=A_{1}=\left\{1\right\}$ and ${\hat{A}}_{0}=A_{0}=\left\{0\right\}$. Then we have ${\hat{P}}_{e}=P_{e}$, which implies that the performance of signal transmission and reception are not affected by channel parameter estimation.

However, when N>1, the bit error probability with inaccurate parameter estimates may be higher than that with true parameters.

Consistent with the parameter settings in Section 3.2, the true parameters are set to $\mu_{\xi }=1.2$ and $\sigma_{\xi }=0.1$ in this subsection. But the estimated parameters may deviate from the true values to some extent. Specifically, we consider the case where ${\hat{\mu }}_{\xi }\in \left\{0.9,1.0,1.1,1.2,1.3,1.4,1.5\right\}$ and ${\hat{\sigma }}_{\xi }\in \left\{0.07,0.08,0.09,0.10,0.11,0.12,0.13\right\}$. For each parameter combination $\left({\hat{\mu }}_{\xi },{\hat{\sigma }}_{\xi }\right)$, the bit error probability induced by the injected Gaussian noise with intensity σ, denoted by ${\hat{P}}_{e}\left({\hat{\mu }}_{\xi },{\hat{\sigma }}_{\xi },\sigma \right)$, is calculated via Equation (10), where σ takes values from 0 to 2 with a step size of 0.01 (similar to Figure 3). The set of the values of σ is denoted by $S_{\sigma }=\left\{0,0.01,0.02,\cdots ,1.99,2\right\}$. Meanwhile, the bit error probability under the true parameters $\mu_{\xi }$ and $\sigma_{\xi }$, i.e., $P_{e}\left(\mu_{\xi },\sigma_{\xi },\sigma \right)$, can be obtained as well, as presented in Section 3.2.

To quantify the impact of parameter estimation sensitivity on system performance, the mean squared difference (MSD) between the bit error probabilities under true parameters and inaccurate estimates is defined as follows:

(17) $$\mathrm{MSD}=\frac{1}{|S_{\sigma }|}\sum_{\sigma \in S_{\sigma }}{\left({\hat{P}}_{e}\left({\hat{\mu }}_{\xi },{\hat{\sigma }}_{\xi },\sigma \right)-P_{e}\left(\mu_{\xi },\sigma_{\xi },\sigma \right)\right)}^{2}.$$

**Figure 4:**
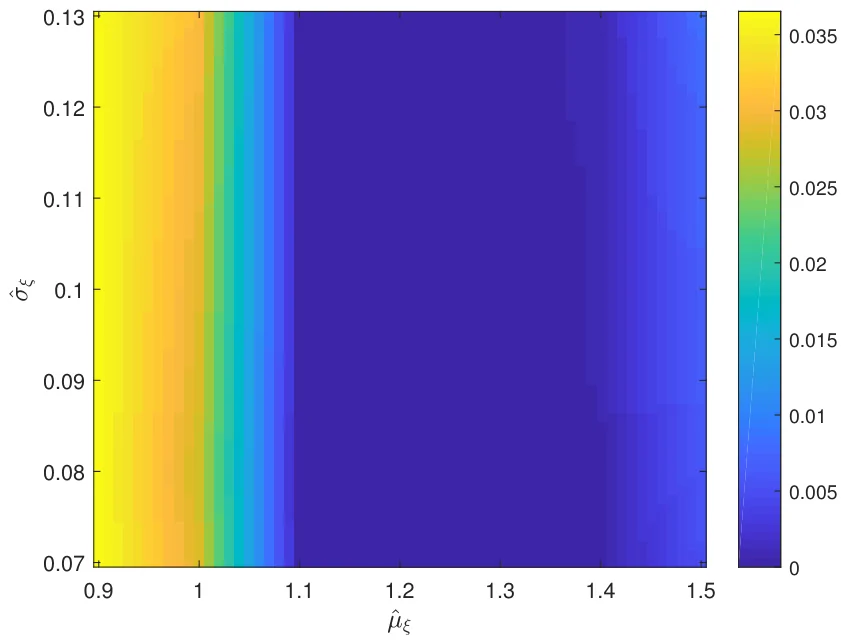
MSD between the bit error probabilities under true parameters $\left(\mu_{\xi },\sigma_{\xi }\right)$ and inaccurate estimates $\left({\hat{\mu }}_{\xi },{\hat{\sigma }}_{\xi }\right)$. The true parameters are $\mu_{\xi }=1.2$ and $\sigma_{\xi }=0.1$. The number of the one-bit ADCs is N=10, with the threshold u=0. The probabilities of the input signal are $P_{0}=P_{1}=0.5$.

For various inaccurate parameter estimates, the MSD in Equation (17) is shown in Figure 4.

**Figure 5:**
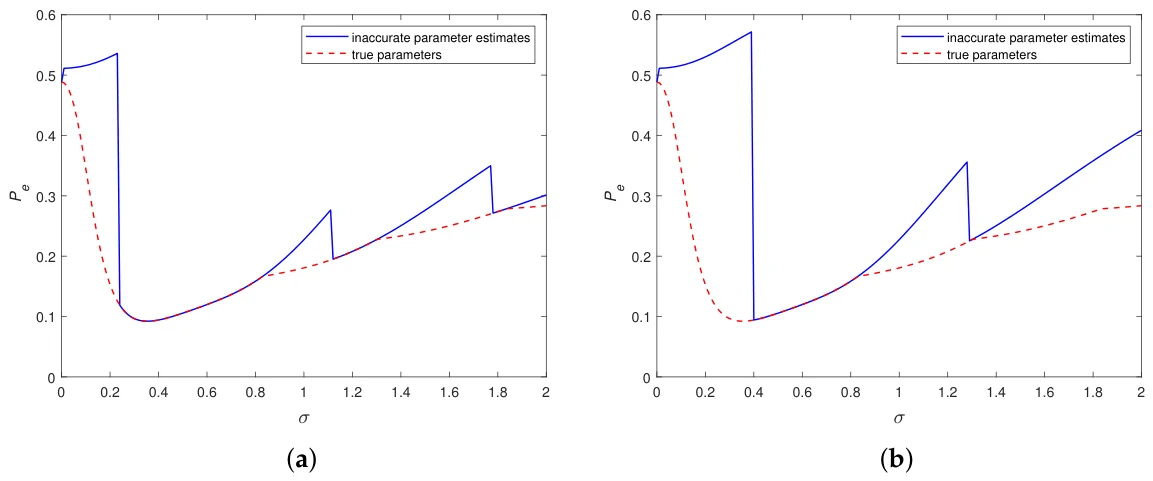
The bit error probability versus the intentionally injected Gaussian noise intensity σ. The dashed line represents the performance under true parameters ($\mu_{\xi }=1.2$, $\sigma_{\xi }=0.1$), whereas the solid line represents the performance under inaccurate parameter estimates: (**a**) ${\hat{\mu }}_{\xi }=1.5$, ${\hat{\sigma }}_{\xi }=0.13$; (**b**) ${\hat{\mu }}_{\xi }=1.6$, ${\hat{\sigma }}_{\xi }=0.14$. Moreover, the number of one-bit ADCs is N=10, the threshold is u=0, and the probabilities of the input signal are $P_{0}=P_{1}=0.5$.

It can be observed from Figure 4 that for ${\hat{\mu }}_{\xi }\in \left[1.1,1.4\right]$, ${\hat{\sigma }}_{\xi }\in \left[0.07,0.13\right]$, the MSD remains nearly zero, which demonstrates that the ML detector given by Equations (6) and (7) exhibits a certain degree of robustness. As long as the parameter estimation deviations, i.e., $|{\hat{\mu }}_{\xi }-\mu_{\xi }|$ and $|{\hat{\sigma }}_{\xi }-\sigma_{\xi }|$, remain within a certain range, inaccurate parameter estimates do not degrade the performance of signal transmission and reception. Especially when the optimal noise intensity $\sigma_{\mathrm{opt}}$ is used, the minimum bit error probability can be achieved for inaccurate parameters. As shown in Figure 5a, for the inaccurate parameters ${\hat{\mu }}_{\xi }=1.5$ and ${\hat{\sigma }}_{\xi }=0.13$, although the bit error probability under inaccurate estimates is considerable larger than that under true parameters at certain noise intensities, the bit error probabilities are equal at the optimal intensity $\sigma_{\mathrm{opt}}$. In this case, the optimal noise intensity is $\sigma_{\mathrm{opt}}\approx 0.35$, and the corresponding bit error probabilities are ${\hat{P}}_{e}\left({\hat{\mu }}_{\xi },{\hat{\sigma }}_{\xi },\sigma_{\mathrm{opt}}\right)=P_{e}\left(\mu_{\xi },\sigma_{\xi },\sigma_{\mathrm{opt}}\right)=0.0923$.

On the other hand, when the parameter estimates deviate significantly from the true values, the performance of signal transmission and reception may be adversely affected to some extent. Considering the scenario where the parameters are estimated to be ${\hat{\mu }}_{\xi }=1.6$ and ${\hat{\sigma }}_{\xi }=0.14$, we obtain the performance curve (solid line) shown in Figure 5b. In this case, the bit error probability substantially differs from that under exact parameter values. In particular, we have $\min_{\sigma \in S_{\sigma }}{\hat{P}}_{e}\left({\hat{\mu }}_{\xi },{\hat{\sigma }}_{\xi },\sigma \right)=0.0943>P_{e}\left(\mu_{\xi },\sigma_{\xi },\sigma_{\mathrm{opt}}\right)=0.0923$.

Owing to the robustness of the ML detector, the impact of inaccurate parameter estimates on the performance of signal transmission and reception is not considered in the subsequent sections. We assume that the parameters of channel noise can be accurately estimated.

## 4. Transmission Performance of Hamming Codes

Based on the results (the probabilities $P_{1|0}$, $P_{0|1}$, or $P_{e}$) of the uncoded signal transmission in the preceding section, we further explore the noise-enhanced performance in the coded case. In this section, the binary Hamming code C(n,k) is taken into consideration. The length of codewords *n* and the number of message bits *k* are determined by $n=2^{l}-1$ and $k=2^{l}-l-1$, where *l* is a positive integer.

For Hamming encoding and decoding, the generator matrix and the parity-check matrix in the systematic form are used. As shown in Figure 1, the source information *m* is first encoded by a Hamming encoder and then transmitted over the additive Gaussian mixture noise channel.

### 4.1. Theoretical Analysis

The weight distribution of the Hamming code is important. It can be applied to calculate some error probabilities. For a binary Hamming code C(n,k), let $A_{i}$ denote the number of codewords of weight *i*, i=0,1,⋯,n. Then $A_{i}$’s are used to form a polynomial $A\left(z\right)=\sum_{i=0}^{n}A_{i}z^{i}$, which is called the weight enumerator. In other words, $A_{i}$ is the coefficient of the term $z^{i}$ in the expansion of the weight enumerator. The weight distribution of the Hamming code is given as follows.

**Lemma 2**  ([33])**.** *The weight enumerator of the binary Hamming code C(n,k) is*

(18) $$A\left(z\right)=\frac{1}{n+1}\left[{\left(1+z\right)}^{n}+n\left(1-z\right){\left(1-z^{2}\right)}^{\frac{n-1}{2}}\right].$$

We assume the a priori probabilities of source bit *m* being 0 and 1 are $P_{0}=P_{1}=0.5$. Based on Lemma 2, we have the following conclusion.

**Lemma 3.**  *If the probabilities of the source bit m are Pr{m=0}=Pr{m=1}=0.5, then the probabilities of the coded bit w stay the same, i.e., Pr{w=0}=Pr{w=1}=0.5.*

The proof is in Appendix D.

In this section, the modulator, the demodulator, the ADCs, and the ML detector depicted in Figure 1 are considered to be the same as those in Section 3. When $P_{0}=P_{1}=0.5$, Lemma 3 guarantees that the results in the uncoded scenario in Section 3, e.g., the optimal noise intensity, can also be used here.

As shown in Figure 1, when the symbols of *r* are determined, the syndrome decoding scheme based on the parity-check matrix of the Hamming code is used to obtain the estimation of codewords. In addition to the standard syndrome decoding, there are some other decoding methods for the Hamming code, such as the minimum of sums decoding [13], the majority bit decoding [13], and even the decoding technique based on artificial neural networks [34]. These decoding methods are not employed in this paper but remain to be examined in future research.

For the binary Hamming code C(n,k) transmitted through the additive Gaussian mixture noise channel and quantized by the ADCs, the performance induced by the injected Gaussian noise can be expressed as follows.

**Theorem 2.**  *If the a priori probabilities are $P_{0}=P_{1}=0.5$, then for the binary Hamming code C(n,k), when the syndrome decoding is applied, the codeword error probability $P_{B}$ is*

(19) $$\begin{array}{cc} P_{B}= & 1-2^{-k}[A_{0}\left(P_{0|0}^{n}+nP_{1|0}P_{0|0}^{n-1}\right)+A_{n}\left(P_{1|1}^{n}+nP_{0|1}P_{1|1}^{n-1}\right)\\ \; & +\sum_{i=1}^{n-1}A_{i}\left(P_{0|0}^{n-i}P_{1|1}^{i}+iP_{0|1}P_{0|0}^{n-i}P_{1|1}^{i-1}+\left(n-i\right)P_{1|0}P_{0|0}^{n-i-1}P_{1|1}^{i}\right)], \end{array}$$

*where $P_{1|0}$ and $P_{0|1}$ are determined by Equations (8) and (9), and $P_{0|0}=1-P_{1|0}$, $P_{1|1}=1-P_{0|1}$. In addition, the information bit error probability $P_{b}$ is lower bounded by*

(20) Pb≥3PB(22l−1−l·2l−1−3·2l−1+l+1)kn2,

*where $n=2^{l}-1$ and $k=2^{l}-l-1$.*

The proof is in Appendix E.

**Remark 2.**  *If the transition probabilities $P_{1|0}$ and $P_{0|1}$ are quite small, there is a high probability that no more than two errors occur during the transmission of a codeword. In this case, the information bit error probability $P_{b}$ will closely approximate the lower bound in Equation (20).*

### 4.2. Results of Transmission Performance

Under identical channel conditions and for the same family of error-correcting codes, a higher code rate may lead to a higher bit error rate. The code rate of a binary Hamming code C(n,k) is

$$R=\frac{k}{n}=\frac{2^{l}-l-1}{2^{l}-1},$$

which monotonically increases with respect to *l*. To achieve a low information bit error probability, the value of *l* should be selected to be as small as possible for the Hamming codes.

In this subsection, a systematic Hamming code C(7,4), i.e., l=3, is used to explore the noise-enhanced effect. Both theoretical and simulation results are presented. For Monte Carlo simulation, $2\times 10^{7}$ information bits are used. It should be mentioned that for other Hamming codes C(n,k), the SR effect maybe exist as well.

**Figure 6:**
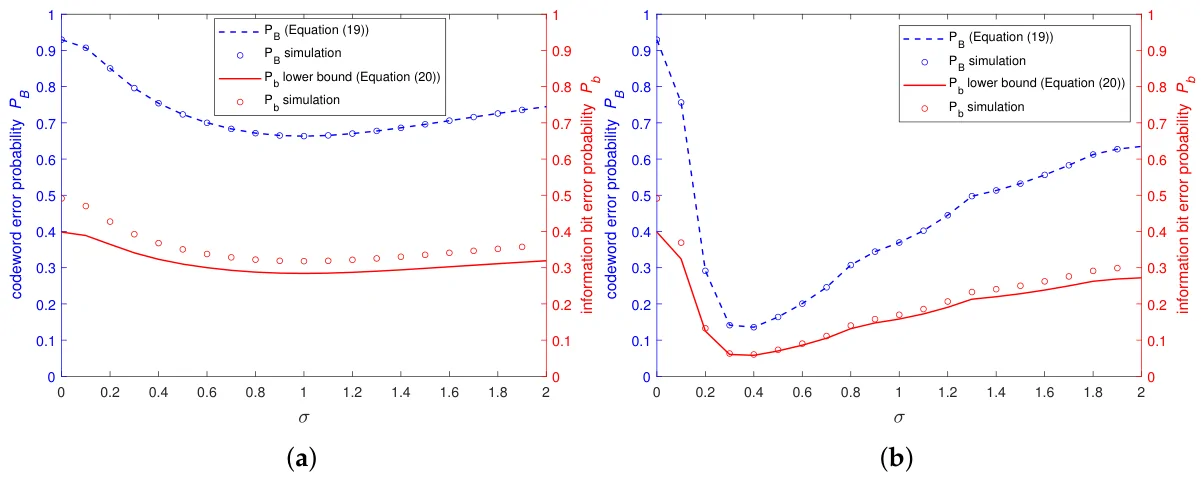
The codeword error probability $P_{B}$ and the information bit error probability $P_{b}$ versus the intentionally injected Gaussian noise intensity σ. The parameters of the additive Gaussian mixture noise in the channel are $\mu_{\xi }=1.2$ and $\sigma_{\xi }=0.1$. Moreover, the threshold of the one-bit ADC is u=0, and the number of ADCs is (**a**) N=1; (**b**) N=10. The dashed lines and the solid lines represent the theoretical results, whereas simulation results are marked by circles.

For a fixed Gaussian mixture noise channel, the performance of Hamming code transmission is clearly demonstrated in Figure 6.

The classical phenomenon of SR is observed from Figure 6. As the intensity of the injected Gaussian noise increases, the error probability decreases to the minimum value and then increases gradually. It means that an appropriate amount of Gaussian noise may aid the transmission and reception of Hamming codes. However, Figure 6a,b show different noise-enhanced performance for two distinct *N*’s. When more one-bit ADCs are used, more information will be retained during quantization. So a larger *N* will lead to a better transmission performance. Such a result is in accordance with that in the uncoded case in Figure 3.

Additionally, the lower bound of the information bit error probability is derived in the case that no more than two errors occur in a Hamming codeword. When there are three or more errors, the probability of such an event scales as $O(P_{e}^{3})$. Note that the channel in this paper is not a binary symmetric one. The expression $O(P_{e}^{3})$ is just an approximation. As shown in Figure 3, when the number N=10, the bit error probability $P_{e}$ remains fairly low for σ∈[0.2,0.6], which suggests a high probability that no more than two errors will occur in a codeword. Equivalently, the $P_{b}$ obtained by simulation is very close to the lower bound for σ∈[0.2,0.6], as shown in Figure 6b.

**Figure 7:**
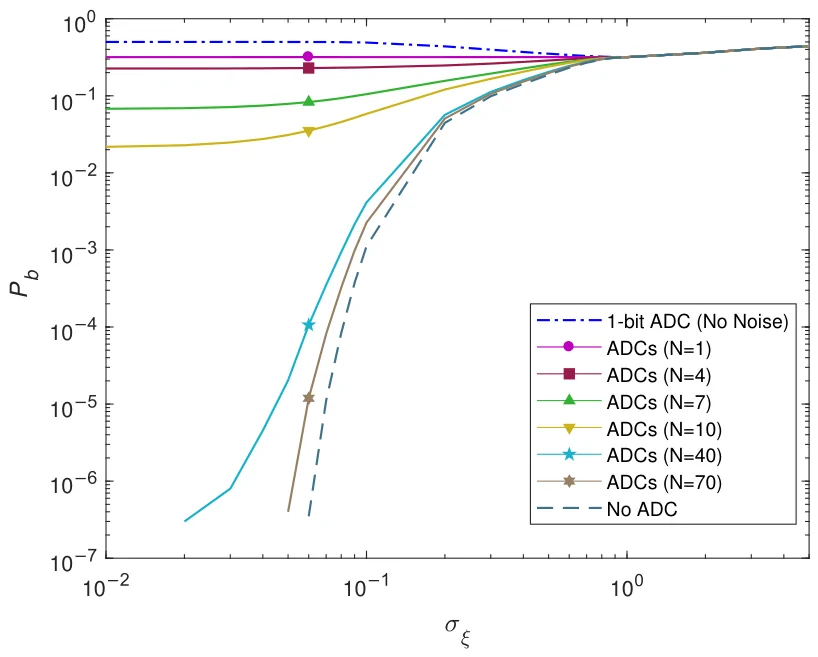
The information bit error probability $P_{b}$ versus the channel noise parameter $\sigma_{\xi }$. The parameter $\mu_{\xi }$ is fixed as $\mu_{\xi }=1.2$, and the threshold of the one-bit ADC is u=0. The top dotted–dashed line represents the performance in the case that no noise is added, i.e., σ=0. The solid lines combined with different markers represent the performance induced by the injected Gaussian noise with optimal intensity. The bottom dashed line represents the performance when no ADC is employed, in which case the channel outputs are directly decided by the ML detector without being quantized.

In Section 3.2, the optimal intensity of the injected Gaussian noise, $\sigma_{\mathrm{opt}}$, which helps to induce the minimum $P_{e}$, was determined (see the markers in Figure 3). Now the Gaussian noise with the optimal intensity is used to enhance the transmission and reception of Hamming codes. For various Gaussian mixture noise channels ($\sigma_{\xi }$ taking various values), the performance of Hamming code transmission is displayed in Figure 7.

It is observed from Figure 7 that all solid lines are located below the dotted–dashed line, which means the injected noise (especially with the optimal intensity) improves the performance of Hamming code transmission and reception. As mentioned above, with the increase in the number *N*, the SR or noise-enhanced effect becomes more and more remarkable. In addition, the optimal detecting and decoding performance is shown by the dashed line in Figure 7. Since no ADC is used, the channel outputs are received and decided by the ML detector without being quantized. In this case, almost all information is retained. So the detector and the decoder perform the best, but with high complexity. Furthermore, under bad channel conditions, e.g., $\sigma_{\xi }>0.9$ in Figure 7, the Hamming codes are corrupted by strong channel noise and produce completely random channel outputs. The transmission performance becomes unsatisfactory even in the case of optimal reception. And certainly in this situation, the SR effect is not available.

Although Hamming coding is applied in this paper, it should be mentioned that when some other coding scheme with a greater error correction capacity (e.g., LDPC codes or turbo codes) is adopted, a more remarkable SR effect can be achieved and the signal transmission performance may be further improved.

## 5. Performance of Speech Signal Transmission

A speech (audio) signal transmitted over the additive Gaussian mixture noise channel is considered as an example in this section. At the same time, the effect of noise enhancing transmission performance is examined.

**Figure 8:**
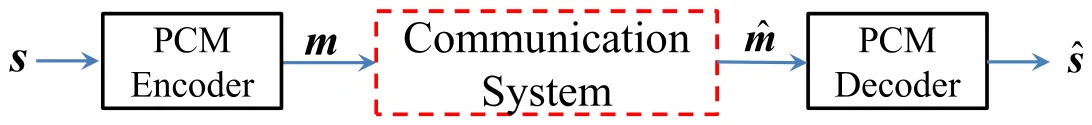
The scheme of speech signal transmission. The communication system module in the dashed-line box is depicted in Figure 1.

Figure 8 shows the block diagram of the process of a speech signal transmission. In this section, all variables appear in vector forms.

At first, a speech signal s is sampled, quantized, and encoded by a pulse code modulation (PCM) encoder. We expect to explore the SR effect during the speech transmission and do not pay much attention to speech signal processing. Just the PCM coding is considered here. Other speech coding techniques can be applied in a similar way.

The PCM encoder produces a binary symbol sequence m, which is encoded into Hamming codes and transmitted over an additive Gaussian mixture noise channel. As investigated in Section 4, after quantization, deciding, and decoding, the bit sequence m^ is obtained. Subsequently, the sequence m^ is passed to the PCM decoder to reconstruct the received speech signal s^.

To measure the perceptual quality of the reconstructed speech signal s^, a widely adopted objective metric, the perceptual evaluation of speech quality (PESQ) [35], and the subjective mean opinion score (MOS) [36] are employed in this section. For the two measures, a larger value indicates better speech quality. Computation of the two measures was presented by Ref. [37]. Particularly, the mapping function which transforms the PESQ of narrowband speech to the MOS has the following form [37],

$$\mathrm{MOS}=0.999+\frac{4.999-0.999}{1+e^{-1.4945\cdot \mathrm{PESQ}+4.6607}},$$

where PESQ ranges from −0.5 to 4.5.

**Figure 9:**
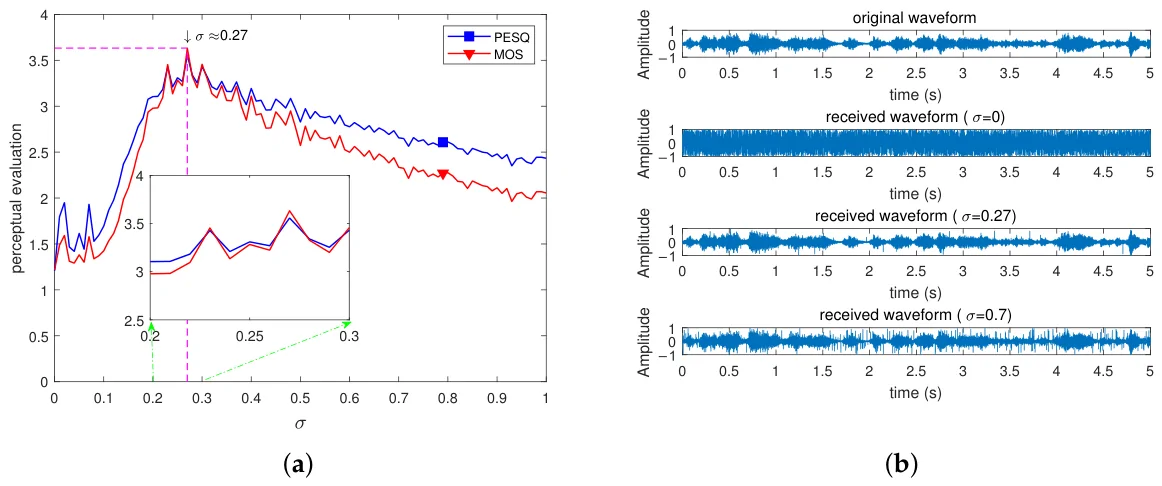
(**a**) The quality evaluation of a reconstructed speech signal; (**b**) waveforms of the original speech signal and the reconstructed speech signal. The parameters of the Gaussian mixture noise in the channel are $\mu_{\xi }=1.2$ and $\sigma_{\xi }=0.1$. Moreover, the threshold of the one-bit ADC is u=0, and the number of ADCs is N=70.

Here, a specific speech signal, which has been sampled with the sampling frequency 8192 Hz, is taken into account. At first, the speech signal is resampled to 8 kHz (narrowband speech) in accordance with ITU-T P.862 [35]. After resampling, the PESQ and the MOS can be obtained based on the original speech signal s and the reconstructed speech signal s^. Specifically, the quality of speech reconstruction enhanced by intentionally injected Gaussian noise is shown in Figure 9.

As seen in Figure 9a, when σ=0, i.e., no Gaussian noise is injected at the receiving end, the MOS is as low as 1.2173, which, according to the MOS rating scale [37], indicates bad quality of the reconstructed speech signal. In contrast, when an appropriate amount of Gaussian noise is introduced (σ≈0.27), the maximum PESQ and MOS are achieved. Particularly, the MOS reaches its maximum value of 3.6329, at which point, the quality of the reconstructed speech signal approaches the good level. These observations imply that the intentionally injected noise may enhance the performance of speech signal transmission and reconstruction. That is to say, the SR effect exists in this scenario. More specifically, Figure 9b displays the received waveform of the reconstructed speech signal. When no noise is injected (σ=0), the received waveform is so chaotic and random that little information can be extracted. Moreover, in the case of a large intensity (σ=0.7), there is some non-negligible deformation by comparing the received waveform with the original one. By contrast, with the aid of a moderate amount of noise (σ≈0.27), just a little distortion appears during the speech signal transmission and reception. And an acceptable reconstructed signal can be obtained. The above-mentioned results indicate the application of the SR effect in speech signal processing and transmission.

## 6. Conclusions and Discussion

In this paper, we pay attention to the noise-enhanced performance of the signal transmission over an additive Gaussian mixture noise channel with one-bit ADCs. The SR or noise-enhanced effect has been revealed in both uncoded and coded cases. When various parameters are considered, the signal transmission performance is shown clearly. Particularly, the optimal intensity of the intentionally injected Gaussian noise is determined. And the minimum error probability is achieved accordingly. Moreover, an example of speech signal transmission is presented, which illustrates the applicability of SR.

As we have mentioned in Section 4, in addition to the Hamming code, there are many other efficient channel codes, e.g., the LDPC code and the Polar code in 5G communications [38,39]. It would be worthwhile to examine the SR effect during the transmission and reception of other codes. As a supplementary result, the SR effect on the transmission performance of LDPC codes is demonstrated in Appendix F.

Further research may focus on some time-varying and fading channels, and whether SR exists should be studied in detail. Moreover, for some practical communication systems, such as MIMO systems, the noise-enhanced effect may be explored as well in the future.

## Figures and Tables

**Figure A1:**
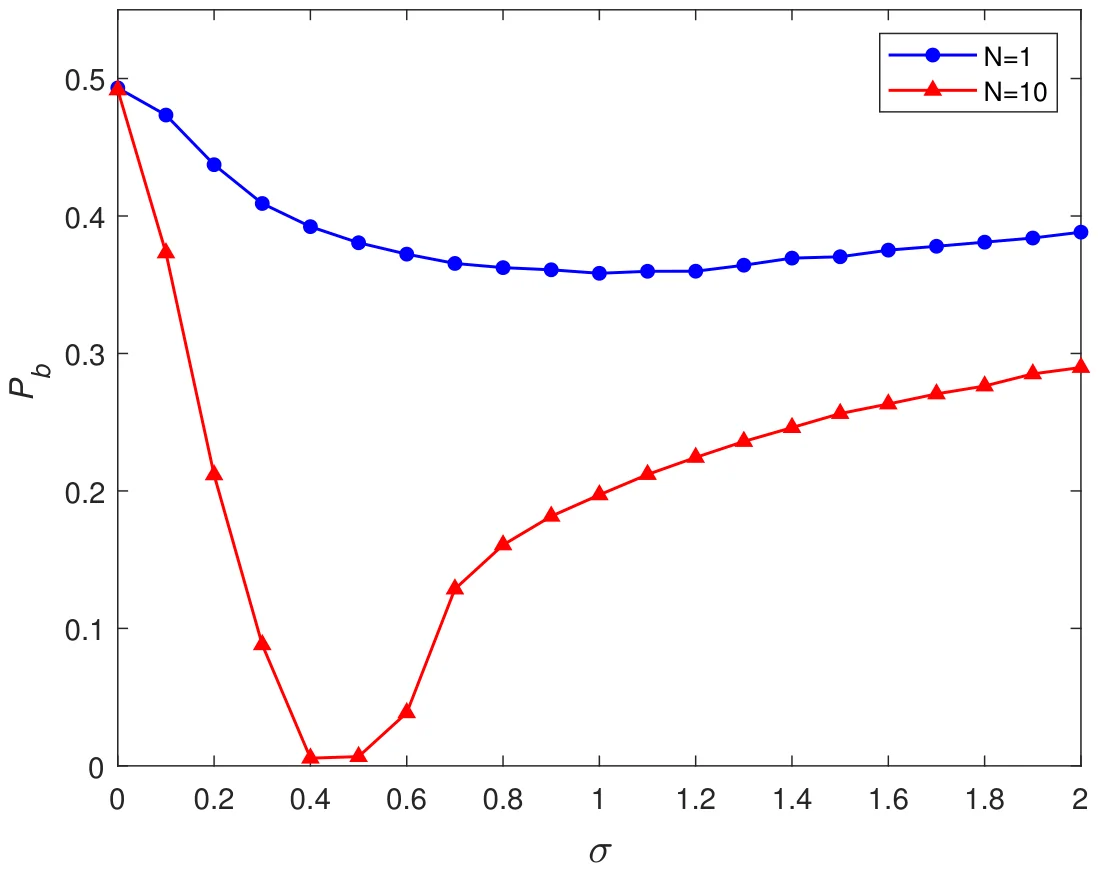
Bit error rate $P_{b}$ versus the intentionally injected Gaussian noise intensity σ. The parameters of the additive Gaussian mixture noise in the channel are $\mu_{\xi }=1.2$, $\sigma_{\xi }=0.1$. The threshold of the one-bit ADC is u=0, and the number of the ADCs is N=1 or N=10.

**Figure A2:**
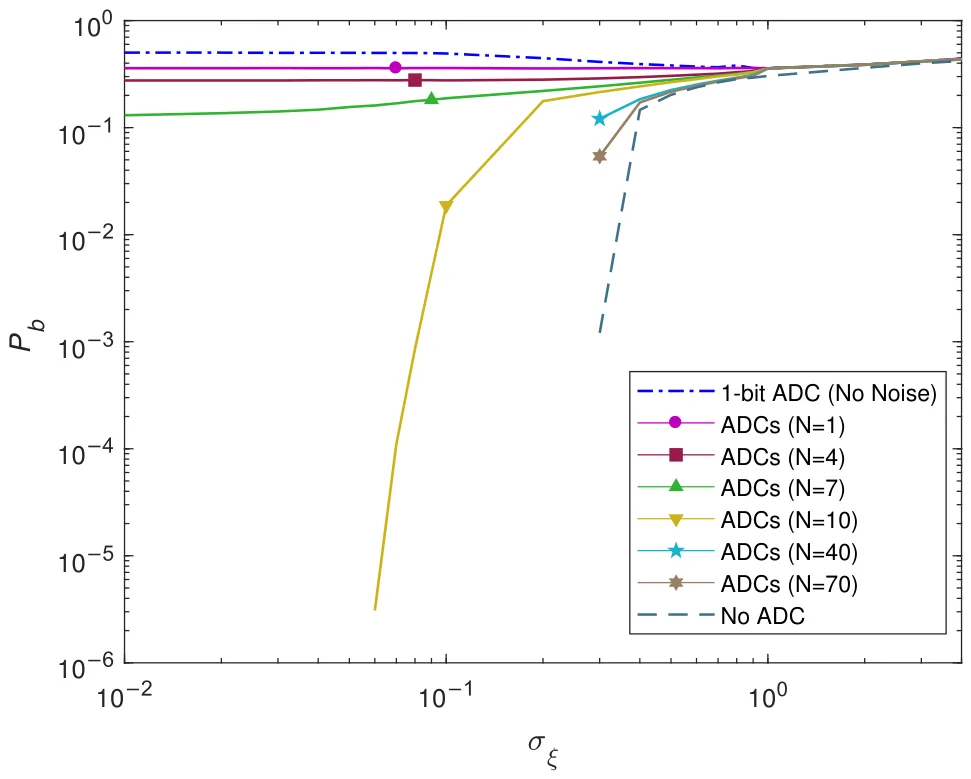
Bit error rate $P_{b}$ versus the channel noise parameter $\sigma_{\xi }$. The top dotted–dashed line represents the LDPC decoding performance when the channel outputs have been quantized by a one-bit ADC. In this case, no noise is intentionally injected into the ADC (i.e., σ=0). The solid lines combined with different markers represent the performance induced by the injected Gaussian noise with optimal intensity. The optimal intensity for each *N* is shown in Figure 3. The bottom dashed line represents the performance of LDPC decoding based on the unquantized channel outputs. It means that no ADC is used in this case.

## Data Availability

The data presented in this study are available on request from the corresponding author due to the restriction.

## Appendix A. Proof of Lemma 1

**Proof of Lemma 1.** Taking the derivative of $P_{e}$ in Equation (11) with respect to $\sigma^{2}$ gives

(A1) $$\begin{array}{cc} \frac{dP_{e}}{d\sigma^{2}} & =\frac{{\left(\sigma_{\xi }^{2}+\sigma^{2}\right)}^{-3/2}}{4\sqrt{2\pi }}\\ \; & \times \left\{P_{0}\left[\left(u+1-\mu_{\xi }\right)\exp\left(-\frac{{\left(u+1-\mu_{\xi }\right)}^{2}}{2(\sigma_{\xi }^{2}+\sigma^{2})}\right)+\left(u+1+\mu_{\xi }\right)\exp\left(-\frac{{\left(u+1+\mu_{\xi }\right)}^{2}}{2(\sigma_{\xi }^{2}+\sigma^{2})}\right)\right]\right.\\ \; & -\left.P_{1}\left[\left(u-1-\mu_{\xi }\right)\exp\left(-\frac{{\left(u-1-\mu_{\xi }\right)}^{2}}{2(\sigma_{\xi }^{2}+\sigma^{2})}\right)+\left(u-1+\mu_{\xi }\right)\exp\left(-\frac{{\left(u-1+\mu_{\xi }\right)}^{2}}{2(\sigma_{\xi }^{2}+\sigma^{2})}\right)\right]\right\}. \end{array}$$

As long as the noise intensity σ gradually increasing from zero leads to a decrease in $P_{e}$, the optimal intensity $\sigma_{\mathrm{opt}}$ must be nonzero and SR must occur. Thus, a sufficient condition for the existence of SR is

$$\lim_{\sigma^{2}\to 0+}\frac{dP_{e}}{d\sigma^{2}}<0.$$

Using Equation (A1), we can obtain Equation (13). □

## Appendix B. Proof of Theorem 1

**Proof of Theorem 1.** For u=0, using Equation (12) or Equation (A1), we have

$$\frac{dP_{e}}{d\sigma^{2}}=\frac{{\left(\sigma_{\xi }^{2}+\sigma^{2}\right)}^{-3/2}}{4\sqrt{2\pi }}\left\{\left(1-\mu_{\xi }\right)\exp\left[-\frac{{\left(1-\mu_{\xi }\right)}^{2}}{2(\sigma_{\xi }^{2}+\sigma^{2})}\right]+\left(1+\mu_{\xi }\right)\exp\left[-\frac{{\left(1+\mu_{\xi }\right)}^{2}}{2(\sigma_{\xi }^{2}+\sigma^{2})}\right]\right\}.$$

When $0<\mu_{\xi }\leq 1$, we have $\frac{dP_{e}}{d\sigma^{2}}>0$. Thus, $P_{e}$ is a monotonically increasing function of $\sigma^{2}$. Then $\sigma_{\mathrm{opt}}^{2}=0$ will induce the minimum $P_{e}$. While $\mu_{\xi }>1$, setting the derivative to 0, we have

$$\sigma_{\xi }^{2}+\sigma^{2}=\frac{2\mu_{\xi }}{\ln\frac{\mu_{\xi }+1}{\mu_{\xi }-1}}.\;$$

Due to the nonnegativity of $\sigma^{2}$, the optimal value must be $\sigma_{\mathrm{opt}}^{2}={\left[\frac{2\mu_{\xi }}{\ln\frac{\mu_{\xi }+1}{\mu_{\xi }-1}}-\sigma_{\xi }^{2}\right]}^{+}$. □

## Appendix C. The Condition for the Existence of SR in the Case of the AWGN Channel and *N* = 1

When $\mu_{\xi }=0$, the Gaussian mixture noise channel becomes Gaussian. Substituting $\mu_{\xi }=0$ into Equation (A1), we have

$$\frac{dP_{e}}{d\sigma^{2}}=\frac{{\left(\sigma_{\xi }^{2}+\sigma^{2}\right)}^{-3/2}}{2\sqrt{2\pi }}\left[P_{0}\left(u+1\right)\exp\left(-\frac{{\left(u+1\right)}^{2}}{2(\sigma_{\xi }^{2}+\sigma^{2})}\right)-P_{1}\left(u-1\right)\exp\left(-\frac{{\left(u-1\right)}^{2}}{2(\sigma_{\xi }^{2}+\sigma^{2})}\right)\right].$$

Case 1: −1≤u≤1. In this case, $\frac{dP_{e}}{d\sigma^{2}}>0$. Thus, $\sigma_{\mathrm{opt}}^{2}=0$ and there is no SR effect.

Case 2: u>1 or u<−1. By solving $\frac{dP_{e}}{d\sigma^{2}}=0$, it is easy to obtain

$$\sigma_{\mathrm{opt}}^{2}={\left[\frac{2u}{\ln\frac{P_{0}\left(u+1\right)}{P_{1}\left(u-1\right)}}-\sigma_{\xi }^{2}\right]}^{+}.$$

Or equivalently, the condition for the existence of SR in this case is $\frac{2u}{\ln\frac{P_{0}\left(u+1\right)}{P_{1}\left(u-1\right)}}>\sigma_{\xi }^{2}$.

## Appendix D. Proof of Lemma 3

**Proof of Lemma 3.** For the binary Hamming code C(n,k), there are $2^{k}$ possible messages, accordingly mapped to $2^{k}$ codewords. Since Pr{m=0}=Pr{m=1}=0.5, each codeword c has the probability $\mathrm{Pr}\left\{c\right\}=2^{-k}$. Then we have

(A2) $$\mathrm{Pr}\left\{w=1\right\}=\sum_{c}\mathrm{Pr}\{c\}\mathrm{Pr}\{1|c\}=2^{-k}\sum_{i=0}^{n}A_{i}\cdot \frac{i}{n}.$$

Note that $A\left(z\right)=\sum_{i=0}^{n}A_{i}z^{i}$. Thus,

(A3) $$\sum_{i=0}^{n}iA_{i}={\left.\frac{dA(z)}{dz}\right|}_{z=1}.$$

By substituting Equation (18) into Equation (A3), we have $\sum_{i=0}^{n}iA_{i}=\frac{n\cdot 2^{n-1}}{n+1}$. Then Equation (A2) can be simplified as $\mathrm{Pr}\left\{w=1\right\}=\frac{n\cdot 2^{n-1}}{2^{k}\cdot n\left(n+1\right)}=\frac{1}{2}$. □

## Appendix E. Proof of Theorem 2

**Proof of Theorem 2.** As the syndrome decoding method is used, the Hamming code C(n,k) is capable of correcting the error patterns with exactly a single error [11]. When no error or just a single error occurs during the transmission of a codeword, we can obtain a correct decoded word. Let c be a codeword with weight *i*; i.e., the numbers of bit ‘1’ and bit ‘0’ in c are *i* and n−i. Correspondingly, the output of the decoder is c^. Since the transition probabilities have been determined by Equations (8) and (9), we have

Pr{c=c^|c}=P0|0n−iP1|1i+iP0|1P1|1i−1P0|0n−i+(n−i)P1|0P0|0n−i−1P1|1i.

Note that the number of codewords with weight *i* is $A_{i}$, and $\mathrm{Pr}\left\{c\right\}=2^{-k}$. Averaged over all codewords, the average probability of codeword correcting is

$$\begin{array}{cc} \; & 2^{-k}\left[A_{0}\left(P_{0|0}^{n}+nP_{1|0}P_{0|0}^{n-1}\right)+A_{n}\left(P_{1|1}^{n}+nP_{0|1}P_{1|1}^{n-1}\right)\right.\\ \; & \;\left.+\sum_{i=1}^{n-1}A_{i}\left(P_{0|0}^{n-i}P_{1|1}^{i}+iP_{0|1}P_{0|0}^{n-i}P_{1|1}^{i-1}+\left(n-i\right)P_{1|0}P_{0|0}^{n-i-1}P_{1|1}^{i}\right)\right]. \end{array}$$

Then Equation (19) can be obtained. To give a lower bound of the information bit error probability, we just consider the case that double errors occur when a codeword is transmitted. The total number of events is n2. All events are equiprobable due to $P_{0}=P_{1}=0.5$. These events can be divided into several disjoint classes as shown in Table A1. **Table A1:** The cases of double errors occurring in a Hamming codeword.

Before Decoding	After Decoding	The Number of Events
two errors in parity-check bits	one error in information bits	n−k2
one error in parity-check bits and one error in information bits	one error in information bits	n−k1·l−11
	two errors in information bits	n−k1·(k−l−11)
two errors in information bits	two errors in information bits	$l\cdot (2^{l-1}-l)$
	three errors in information bits	k2−l·(2l−1−l) According to the results in Table A1, we may calculate the average number of errors in information bits. That is,

n−k2+n−k1·l−11+2[n−k1·(k−l−11)+l·(2l−1−l)]+3[k2−l·(2l−1−l)]n2=3(22l−1−l·2l−1−3·2l−1+l+1)n2.

Subsequently, the lower bound in Equation (20) can be achieved. □

## Appendix F. Transmission Performance of LDPC Codes

In this section, we will show the SR effect on the transmission and reception of LDPC codes. Like Hamming codes, LDPC codes are also block codes. More importantly, the LDPC coding scheme has been applied in 5G communications [38,39]. According to the DVB-S2 standard [40], we consider that the length of one LDPC codeword is 64,800. Particularly, an LDPC code with rate 1/2 is used in this section, which means 32,400 information bits should be used to produce one codeword. In this case, a parity-check matrix with size 32,400 × 64,800 is adopted.

Note that the model of the communication system in Figure 1 is still used here, except that the Hamming encoder and decoder are replaced by the LDPC encoder and decoder. For LDPC decoding, the Min-Sum algorithm [41] is applied. Additionally, 10 iterations of iterative decoding are performed for each received sequence during Monte Carlo simulation.

When LDPC codes are transmitted through an additive Gaussian mixture noise channel with one-bit ADCs, the performance induced by the injected Gaussian noise is shown in Figure A1.

Similar to Figure 6, Figure A1 shows that, under certain conditions, the minimum $P_{b}$ is achieved at some nonzero σ for the LDPC coding scheme. That is to say, the SR effect exists during the transmission and reception of LDPC codes as well.

Moreover, as the parameter of the Gaussian mixture noise channel changes, the corresponding transmission performance of LDPC codes is shown in Figure A2.

As shown in Figure A2, when the channel condition is not bad (e.g., $\sigma_{\xi }<1$), noise can help to enhance the transmission of LDPC codes. Especially for the cases with a large number of one-bit ADCs, the bit error rate $P_{b}$ may approach zero. These results validate that, in some nonlinear communication systems, the SR effect is applicable in different error correction coding schemes.
